# Impact of childbirth mode and interaction of multiple factors on pelvic floor function: a comprehensive analysis of postpartum muscle strength indicators

**DOI:** 10.3389/fgwh.2026.1786977

**Published:** 2026-05-13

**Authors:** Gangwei Wang, Huiling Zhang, Xiaohui Yang, Yuebo Yang, Minjuan Ye, Yifen Li

**Affiliations:** 1 Emergency Department, the Third Affiliated Hospital of Sun Yat-sen University, Guangzhou, Guangdong, China; 2 Department of Gynecology, the Third Affiliated Hospital of Sun Yat-sen University, Guangzhou, Guangdong, China

**Keywords:** biofeedback stimulation, cesearean delivery, childbirth method, pelvic floor dysfunction, pelvic floor function screening

## Abstract

**Purpose:**

Based on electrophysiological assessment data of pelvic floor muscles from 1,781 women, this study systematically analyzes the association patterns among obstetric history, anthropometric indicators, and pelvic floor function through hierarchical regression modeling, aiming to provide a novel perspective for precise risk assessment.

**Methods:**

A retrospective analysis was conducted on data from 1,781 adult women, including age, height, weight, BMI, childbirth history, and pelvic floor screening results. Participants were grouped based on pelvic floor screening scores (above or below 80) and childbirth method (vaginal childbirth or cesarean section). Multiple linear regression was used to analyze risk factors for pelvic floor dysfunction, while *t*-tests, analysis of covariance, and Mann–Whitney *U*-tests were employed to assess the significance of differences between groups.

**Results:**

Patients were divided into two groups based on pelvic floor screening scores (≥80 and <80). There were statistically significant differences between the <80 group and ≥80 group in age, weight, and BMI (age: 38.73 ± 9.95 years vs. 37.03 ± 8.27 years, weight: 57.26 ± 16.63 kg vs. 55.69 ± 7.22 kg, BMI: 22.67 ± 6.72 vs. 21.88 ± 2.56 kg/m², all *p* < 0.05). After adjusting for age, weight, height, and BMI, differences in time since the last childbirth to the current screening were also observed (7.20 ± 9.69 years vs. 4.78 ± 7.99 years, *p* < 0.05). Significant differences in weight were noted between the vaginal childbirth and cesarean section groups (56.32 ± 8.8 kg, *P* < 0.05 vs. 59.59 ± 30.50 kg, *P* < 0.05). After adjusting for the effects of age, weight, height, and BMI, the two groups showed statistically significant differences in the total score (cesarean section 66.13 ± 14.71 vaginal delivery 63.01 ± 16.69, *p* = 0.021), pre-resting stage score (cesarean section 51.28 ± 27.27, vaginal delivery 62.47 ± 25.04, *p* = 0.000), fast-twitch (Type II fibers) stage score (cesarean section 78.89 ± 18.21, vaginal delivery 72.76 ± 22.38, *p* = 0.001), slow-twitch (Type I fibers) stage score (cesarean section 60.93 ± 25.39, vaginal delivery53.21 ± 26.55, *p* = 0.000), post-resting stage score (cesarean section 53.21 ± 26.55, vaginal delivery 60.93 ± 25.39, *p* = 0), and time from the last childbirth to the current screening (cesarean section 7.27 ± 10.04, vaginal delivery 5.03 ± 6.46, *p* = 0.001). Pelvic floor screening total score was negatively correlated with the time since the last childbirth (*B* = −0.233, Beta = −0.135, *p* = 0.013) and number of deliveries (*B* = −1.511, Beta = −0.078, *p* = 0.022), while positively correlated with height (*B* = 1.447, Beta = 0.437, *p* = 0.043).

**Conclusion:**

Multiple deliveries and longer inter-delivery intervals cumulatively impair pelvic floor function. Individualized rehabilitation should prioritize women with shorter stature, older age, or multiple births, alongside promoting a healthy BMI rather than weight loss alone. Postpartum training should be mode-tailored: activating after vaginal birth, relaxing after cesarean.

## 1 Introduction

Pelvic floor dysfunction disorder (PFD) is a common clinical condition that can lead to symptoms such as stress urinary incontinence, bowel dysfunction, or sexual dysfunction, closely related to factors such as childbirth injury, decreased estrogen levels, and obesity (1, 2), significantly impacting patients’ quality of life. Although the incidence and prevalence of PFD have not been systematically investigated, it is estimated that nearly 50% of women experience some form of pelvic floor dysfunction symptoms, with only 10%–20% seeking treatment. With population aging, the prevalence of PFD in women is expected to increase by approximately 50% by 2,050 (3, 4).

The core pathological mechanism of PFD involves coordinated damage to the pelvic floor muscle groups (Type I/II muscle fibers). Slow-twitch (Type I) muscle fibers account for about 70% of the pelvic floor muscle fibers, are slow-contracting and fatigue-resistant, mainly distributed in the deep pelvic floor muscles, responsible for “continuously supporting” organs, maintaining organ position, preventing prolapse, maintaining resting tension, ensuring closure of the urethra, vagina, and anus, as well as supporting posture and intra-abdominal pressure. Fast-twitch (Type II) muscle fibers account for about 30% of the pelvic floor muscle fibers, are fast-contracting and easily fatigued, mainly distributed in the superficial pelvic floor muscles, responsible for “rapidly opening and closing” channels during sudden efforts like coughing, sneezing, jumping, closing the pelvic floor hiatus, and also controlling the initiation and termination of urination and defecation, affecting vaginal tightness and sexual pleasure (5, 6). Current methods for assessing pelvic floor function include pelvic floor ultrasound, MRI, electromyography, and perineometers (7–10). The biofeedback-assisted pelvic floor muscle function training (Biofeedback-Assisted Pelvic Floor Muscle Training, BAPFMT) in this study uses surface electromyography (EMG) technology, combined with a vaginal probe and biofeedback, to activate the muscles electrically. In recent years, BAPFMT has become increasingly popular because it allows both patients and therapists to observe correct contractions of the pelvic floor muscles (PFM), promoting neuromuscular learning or re-adaptation during intervention. This technique has been extensively studied in the treatment of pelvic floor dysfunction, including urgency or frequency of urination, urinary incontinence, bowel disorders, and pain disorders (11, 12). However, there are fewer studies on pelvic floor function screening, and existing research often focuses on single-factor analysis, lacking a systematic exploration of multi-dimensional interactions, making it difficult to fully reveal the complexity of risk-protection mechanisms. This study is based on electrophysiological assessment data of pelvic floor muscles from 1,781 women, using hierarchical regression modeling to systematically analyze the association patterns among obstetric history, anthropometric indicators, and pelvic floor function, providing a new perspective for precise risk assessment.

## 2 Methods

### 2.1 Data source

Data from 1,781 female pelvic floor screening in the gynecological ward and outpatient department of our hospital from 2019 to 2025 were included. Inclusion criteria: (1) Age ≥18 years; (2) Subjects undergoing pelvic floor function screening due to symptoms such as urinary incontinence, frequent urination, constipation, sexual dysfunction, and pelvic pain. Exclusion criteria: (1) History of pelvic malignant tumors or pelvic radiotherapy; (2) Neurogenic bladder or intestinal dysfunction; (3) Severe cardiopulmonary insufficiency; (4) Pregnancy; (5) History of pelvic surgery (unrelated to PFD). Demographic characteristics (age, BMI, pregnancy history, etc.) were collected.

### 2.2 Assessment tools and methods

The pelvic floor screening was conducted using a biofeedback stimulator (MLD B4Splus, Nanjing, China), with the instrument's built-in “biofeedback software” set to “standard screening” mode. Before treatment, the subjects emptied their bladders and lay supine. A disposable vaginal electrode was placed inside the vagina, and four electrode pads were attached to the abdominal surface, two on the anterior superior iliac spine and two beside the navel.

#### 2.2.1 Warm-up phase

Patients contracted their pelvic floor muscles according to voice prompts, which played instructions such as “quick contraction and release, hold the contraction, relax and release.” The warm-up phase primarily taught patients how to contract their pelvic floor muscles, playing templates and voice prompts for rapid contractions and holding contractions. Once familiar, patients could click “Start Screening” to enter the formal screening phase.

#### 2.2.2 Formal phase

The instrument's display showed curves for the pelvic floor muscles and abdominal muscles. The upper part of the curve displayed the contraction of the pelvic floor muscles, while the lower part showed the amplitude of the abdominal muscles. If the abdominal muscles participated when the pelvic floor muscles contracted, the amplitude of the abdominal muscles would increase.

#### 2.2.3 Standard screening process

The standard screening consisted of four stages: pre-rest phase (10 s), fast-twitch muscle (Type II fiber) phase (5 times 33-second rapid contractions), slow-twitch muscle (Type I fiber) phase (5 times slow contractions), and post-rest phase (10 s), totaling 2 min and 35 s (155 s).

#### 2.2.4 Screening results and report interpretation

After the screening, the system automatically displayed the results, showing scores for each of the four stages: pre-rest phase, fast-twitch muscle (Type II fiber) phase, slow-twitch muscle (Type I fiber) phase, and post-rest phase, culminating in a total score. According to the manual and previous research, a total score of 80 was considered passing. The scoring significance of the four stages is as follows (13, 14):

1. Pre-rest Phase: Baseline electromyography value (normal 2–4 μV), reflecting the recovery of the pelvic floor muscles before the test. This phase assessed muscle relaxation ability by measuring muscle resting tension (average) and stability (variability).
2. Slow-Twitch Muscle Function: Electromyographic stability during sustained contraction for 60 s, reflecting endurance, with high average and low variability scores.
3. Fast-Twitch Muscle Function: Reflecting muscle explosive power, with high maximum values and short rise/recovery times scoring highly.
4. Post-rest Phase: Speed of electromyographic recovery after contraction, reflecting the recovery of the pelvic floor muscles after the test. This phase assessed muscle relaxation ability by measuring muscle resting tension (average) and stability (variability).

### 2.3 Statistical analyses

Statistical analyses were performed using SPSS Statistics (version 26.0; IBM Corp., Armonk, NY, USA). Continuous variables are presented as mea*n* ± standard deviation (SD). The Kolmogorov–Smirnov test was used to assess the normality of data distributions. For two-group comparisons of normally distributed data, independent samples *t*-tests were employed. For non-normally distributed data, the Mann–Whitney *U*-test was used. When appropriate, analysis of covariance (ANCOVA) was applied to adjust for potential confounding variables. Multivariate Analysis: Multivariate linear regression analysis was conducted to evaluate the independent associations between various factors and continuous outcomes. A *P*-value < 0.05 was considered statistically significant.

## 3 Research results

### 3.1 Patient demographics and total screening score

Patients were divided into two groups based on a passing score of 80 on the total screening score: ≥80 group and <80 group. There were statistically significant differences between the two groups in age, weight, and BMI. The <80 group (age: 38.73 ± 9.95 years, weight: 57.26 ± 16.63 kg, BMI: 22.67 ± 6.72 kg/m²) had significantly higher age, weight, and BMI compared to the other group (age: 37.03 ± 8.27 years, weight: 55.69 ± 7.22 kg, BMI: 21.88 ± 2.56 kg/m²; all *p* < 0.05).

**Figure 1:**
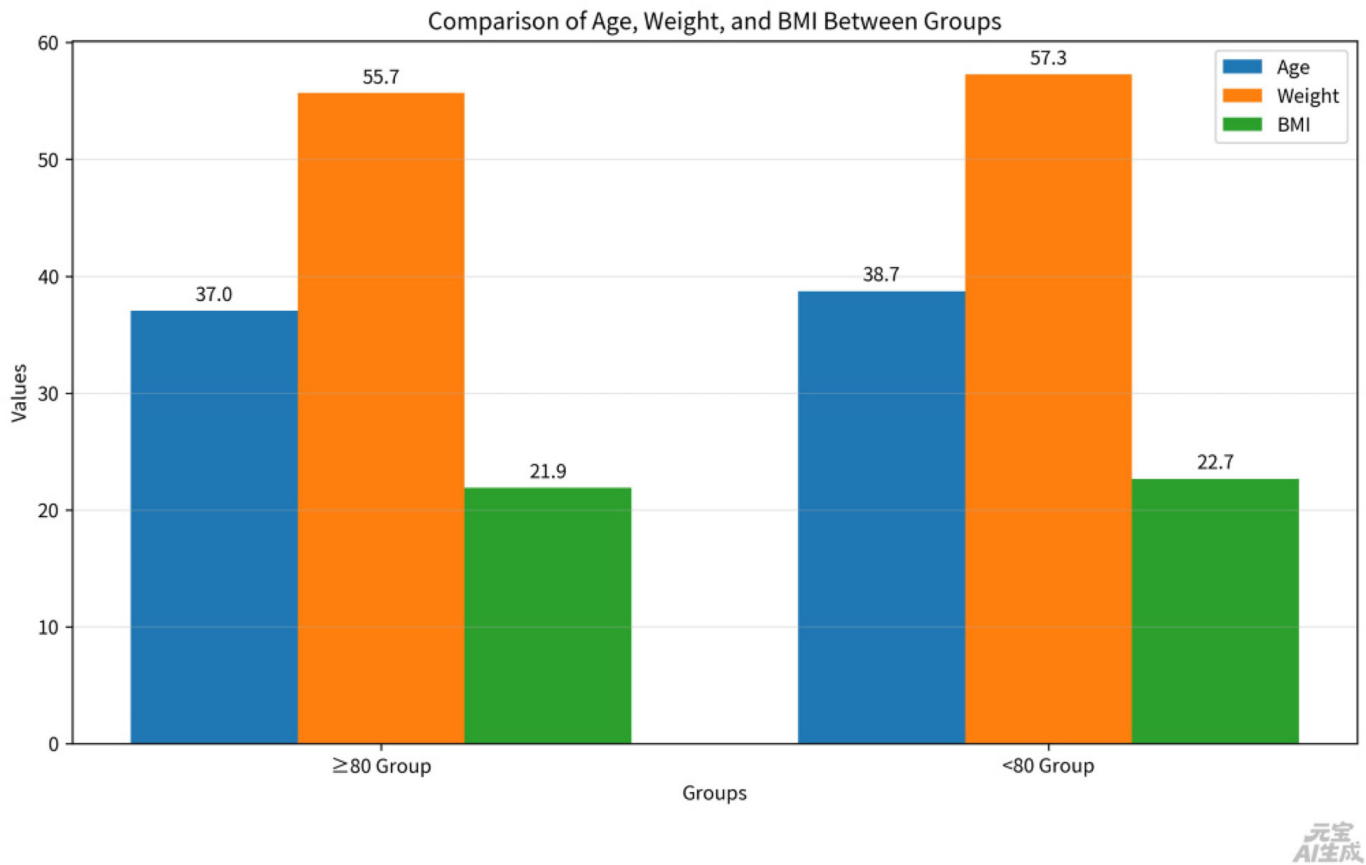
Characteristics between ≥80 group and <80 group.

After adjusting for the effects of age, weight, height, and BMI, there was also a difference in the time from the last childbirth to the current screening between the two groups. The mean interval for the <80 group was 7.20 ± 9.69 years, while that for the other group was 4.78 ± 7.99 years (*p* < 0.05) see Figure 1 and Table 1.

**Table 1:** Comparison of characteristics between ≥80 group and <80 group.

Characteristic	≥80 Group (*n* = 277)	<80 Group (*n* = 1,504)	*P* value
Age (years)	37.03 ± 8.27	38.73 ± 9.95	0.007
Weight (kg)	55.69 ± 7.22	57.26 ± 16.63	0.010
Height (cm)	159.49 ± 4.99	158.92 ± 5.04	0.083
BMI (kg/m²)	21.88 ± 2.56	22.67 ± 6.72	0.001
Time since last childbirth (years)	4.78 ± 7.99	7.20 ± 9.69	0.028

Among the patients who provided detailed obstetric history, there were 925 cases. The Mann–Whitney test indicated that there were no statistically significant differences between the two groups in terms of the number of pregnancies, deliveries, miscarriages, inductions, and newborn weights (*p* > 0.05).

### 3.2 Relationship between childbirth mode and pelvic floor screening scores

**Figure 2:**
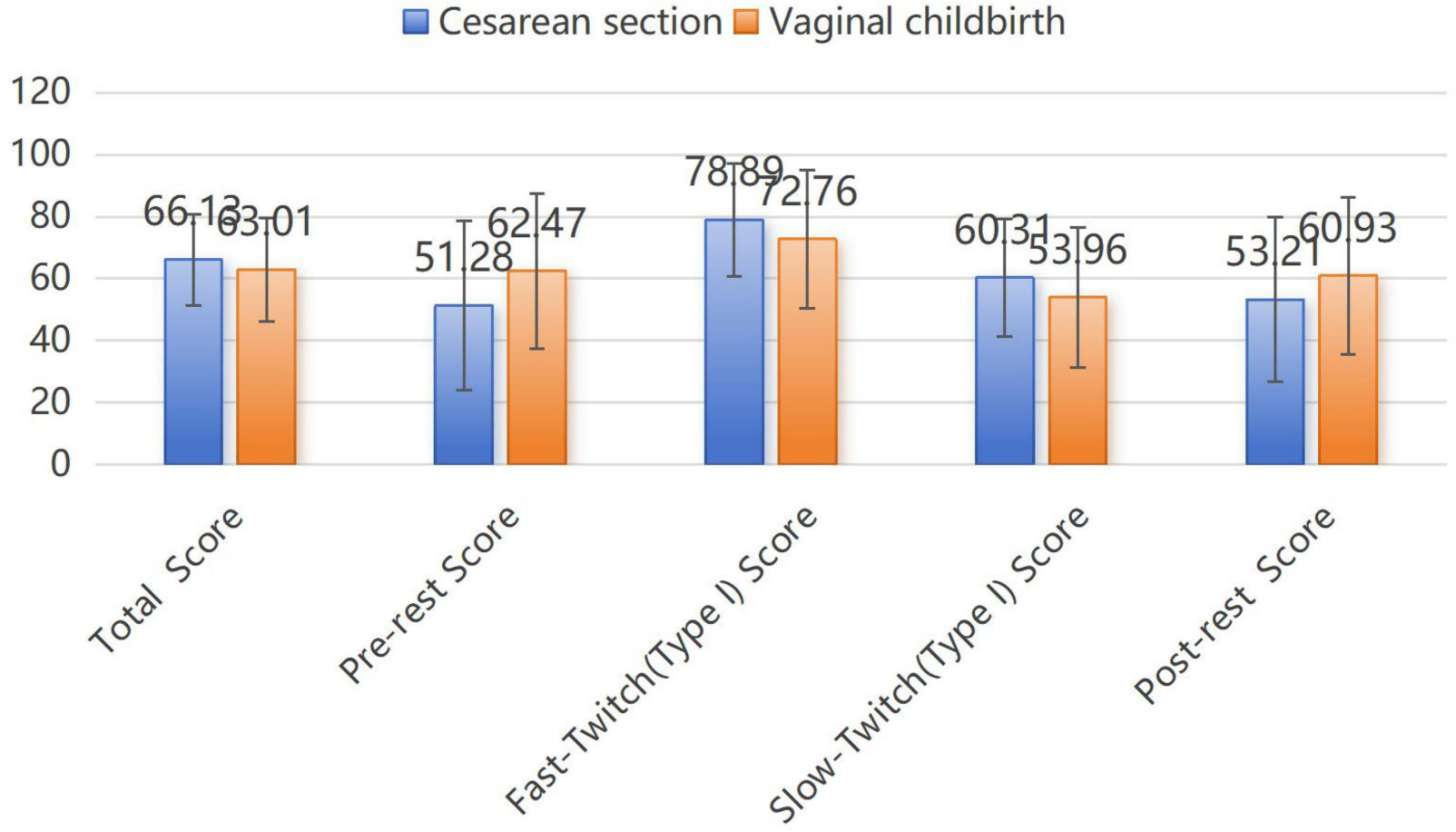
Pelvic floor screening scores between cesarean section and vaginal childbirth groups.

There were 925 patients with detailed obstetric history, of whom 745 had vaginal deliveries and 180 had cesarean sections. The cesarean section group had a significantly higher weight compared to the vaginal childbirth group (59.59 ± 30.50 kg vs. 56.32 ± 8.8 kg, *P* < 0.05), as detailed in Table 2. There were no significant differences between the two groups in terms of the number of deliveries, pregnancies, miscarriages, inductions, and newborn weights (*p* > 0.05). After adjusting for the effects of age, weight, height, and BMI, the two groups showed statistically significant differences in the total score (cesarean section 66.13 ± 14.71 vaginal delivery 63.01 ± 16.69, *p* = 0.021), pre-resting stage score (cesarean section 51.28 ± 27.27, vaginal delivery 62.47 ± 25.04, *p* = 0.000), fast-twitch (Type II fibers) stage score (cesarean section 78.89 ± 18.21, vaginal delivery 72.76 ± 22.38, *p* = 0.001), slow-twitch (Type I fibers) stage score (cesarean section 60.93 ± 25.39, vaginal delivery 53.21 ± 26.55, *p* = 0.000), post-resting stage score (cesarean section 53.21 ± 26.55, vaginal delivery 60.93 ± 25.39, *p* = 0), and time from the last childbirth to the current screening (cesarean section 7.27 ± 10.04, vaginal delivery 5.03 ± 6.46, *p* = 0.001) see Figure 2 and Table 3.

**Table 2:** Comparison of characteristics between vaginal childbirth and cesarean section groups.

Characteristic	Vaginal childbirth (*n* = 745)	Cesarean section (*n* = 180)	*P* value
Age (years)	36.91 ± 9.65	36.18 ± 6.77	0.234
Weight (kg)	56.32 ± 8.8	59.59 ± 30.50	0.012
Height (cm)	159.25 ± 6.42	159.04 ± 4.83	0.626
BMI (kg/m²)	22.97 ± 23.71	23.49 ± 11.41	0.668

**Table 3:** Comparison of pelvic floor screening scores between cesarean section and vaginal childbirth groups.

Item	Group	Mean ± SD	*P* value
Total Score	Cesarean section	66.13 ± 14.71	0.021
	Vaginal childbirth	63.01 ± 16.69	
Pre-rest Score	Cesarean section	51.28 ± 27.27	0.000
	Vaginal childbirth	62.47 ± 25.04	
Fast-Twitch (Type II) Score	Cesarean section	78.89 ± 18.21	0.001
	Vaginal childbirth	72.76 ± 22.38	
Slow-Twitch (Type I) Score	Cesarean section	60.31 ± 18.99	0.001
	Vaginal childbirth	53.96 ± 22.56	
Post-rest Score	Cesarean section	53.21 ± 26.55	0.000
	Vaginal childbirth	60.93 ± 25.39	
Time since last childbirth (years)	Cesarean section	7.27 ± 10.04	0.001
	Vaginal childbirth	5.03 ± 6.46	

Among the predictors, height (*B* = 1.447, SE = 0.712, *β* = 0.437, *t* = 2.031, *p* = 0.043), time (*B* = −0.233, SE = 0.094, *β* = −0.135, *t* = −2.489, *p* = 0.013), and delivery times (*B* = −1.511, SE = 0.658, *β* = −0.078, *t* = −2.295, *p* = 0.022) were significantly associated with the total assessment score. Specifically, a one-unit increase in height was associated with a 1.447-point increase in the score (*p* < 0.05); a one-unit increase in time or delivery times was associated with a 0.233-point or1. 511-point decrease in the score, respectively (both *p* < 0.05). In contrast, weight (*p* = 0.130), age (*p* = 0.084), and BMI (*p* = 0.128) did not show significant associations with the score after adjusting for other variables see Table 4.

**Table 4:** Linear regression analysis of factors associated with pelvic floor screening scores.

	B	SE	Beta	t	*p*	95%CI	
						Lower	Upper
Time since last childbirth (years)	−0.233	0.094	−0.135	−2.489	0.013	−0.417	−0.049
Number of Deliveries	−1.511	0.658	−0.078	−2.295	0.022	−2.803	−0.219
Height (cm)	1.447	0.712	0.437	2.031	0.043	0.048	2.845
Age (years)	−0.177	0.102	−0.099	−1.728	0.084	−0.378	0.024
Weight (kg)	−1.501	0.989	−1.408	−1.517	0.130	−3.442	0.441
BMI (kg/m²)	3.939	2.587	1.378	1.523	0.128	−1.138	9.015

## 4 Discussion

Pelvic floor muscle training (PFMT) has been proven effective in preventing pelvic floor weakness, which manifests as urinary incontinence (UI) and pelvic organ prolapse (POP). However, studies indicate that healthcare providers still have limited awareness of PFMT. Many are unfamiliar with the PERFECTR method, the Modified Oxford Grading Scale, slow and fast contractions, and do not know how to assess pelvic floor screening results (15).

This study combined pelvic floor screening results with factors such as mode of delivery, inter-delivery interval, BMI, and age. It found that the total pelvic floor screening score was negatively correlated with both the time since the last delivery and the number of deliveries, while height was positively correlated with the total score. Patients with a failing total score (<80 points) were older, had higher body weight, higher BMI, and a longer interval since their last delivery. This suggests that a greater number of deliveries and longer intervals between deliveries may exert a cumulative and amplifying negative effect on pelvic floor function over time, causing progressive damage to the muscular and structural integrity of the pelvic floor. Moreover, the capacity for pelvic floor recovery appears diminished in older parturients. The total screening score was positively correlated with height but showed no significant association with body weight or BMI, a finding that differs from previous research (1, 16, 17). This may be because increased height can reduce the load-bearing pressure per unit area of the pelvic floor muscles, enhancing the explosive power of Type II fast-twitch fibers and the endurance of Type I slow-twitch fibers through mechanical leverage advantages. The relationship between weight, BMI, and human health is complex, and their impact on pelvic floor function may not be a simple linear negative correlation. Compared to BMI, body roundness index (BRI), conicity index (CI), waist-to-height ratio (WHtR), body fat percentage, waist circumference (WC), waist-to-hip ratio (WHR), and relative fat mass (RFM) may be more effective in diagnosing urinary incontinence and pelvic floor muscle distress, revealing that body composition (fat/muscle ratio) reflects metabolic status better than weight alone (18, 19). This finding provides a new target for personalized intervention—optimizing fat distribution may be more effective than weight loss.

Previous studies have mostly suggested that women who have had vaginal deliveries are more likely to experience pelvic floor dysfunction than those who have had cesarean sections, with cesarean sections potentially avoiding acute tearing of muscle fibers and possibly offsetting some of the cumulative damage to pelvic floor function from multiple deliveries (20–22). Our study shows the cesarean group scored higher in total, fast- and slow-twitch pelvic floor function, but lower in pre- and post-rest scores, indicating potential resting muscle hypertonicity. The elevated resting tension may stem from disuse atrophy and neural dysregulation over time, rather than acute injury. This highlights a need for early relaxation-focused therapy rather than just strength training to prevent chronic pelvic pain.

Our study suggests that clinical interventions need to be individualized. Studies have shown that exercise in the first year after childbirth can reduce pelvic pain and stress urinary incontinence (23). Therefore, women should be encouraged to engage in low-impact exercise as soon as possible after childbirth, with individual rehabilitation files established for each mother. For vaginal childbirth patients, pelvic floor function training may focus on activation and strengthening, while for cesarean section patients, the focus may be on relaxation and spasm relief.

This study has several limitations. First, its retrospective design restricted access to more detailed obstetric history from medical records, making it difficult to conduct in-depth analyses of the associations with pelvic floor dysfunction. Second, the cesarean section group was not further stratified into elective cesarean delivery and emergency cesarean delivery. Future research could incorporate physiological indicators such as “muscle fiber type” and “resting electromyographic variability” and combine multidimensional data to enhance the assessment of pelvic floor muscle function. Prospective studies that integrate electromyography with pelvic ultrasound and clinical outcome measures will be key to validating and extending these findings.

## 5 Conclusion

Multiple deliveries and longer inter-delivery intervals cumulatively impair pelvic floor function. Individualized rehabilitation, prioritizing women with shorter stature, older age, or multiple births, alongside healthy BMI management, is essential. Postpartum training should be tailored to delivery mode: activation for vaginal birth, relaxation for cesarean.

## Data Availability

The raw data supporting the conclusions of this article will be made available by the authors, without undue reservation.
